# Intracranial Complications Following Acute Rhinosinusitis in a Pediatric Patient Requiring Surgery: A Case Report

**DOI:** 10.7759/cureus.101057

**Published:** 2026-01-07

**Authors:** Takato Sudo, Takefumi Kamakura, Takeshi Tsuda, Yasuo Mishiro

**Affiliations:** 1 Department of Otorhinolaryngology-Head and Neck Surgery, Osaka City General Hospital, Osaka, JPN; 2 Department of Otorhinolaryngology, Osaka University, Suita, JPN

**Keywords:** endoscopic sinus surgery, intracranial abscess, neurosurgery, pediatric sinusitis, streptococcus intermedius

## Abstract

Intracranial complications secondary to acute sinusitis in pediatric patients are rare but potentially life-threatening. Initially, the symptoms are nonspecific, which can result in a delayed diagnosis. If a patient presents with persistent fever and severe headache, an intracranial complication should be suspected and promptly investigated using imaging techniques such as contrast-enhanced computed tomography (CT) and magnetic resonance imaging (MRI). Here, we describe a case in which both a frontal epidural abscess and an interhemispheric subdural empyema developed following acute rhinosinusitis. The patient was a seven-year-old girl experiencing a persistent headache and fever for 10 days. Despite treatment with intravenous antibiotics, the symptoms did not improve. Blood cultures revealed *Streptococcus intermedius* infection. CT and contrast-enhanced MRI revealed both a left frontal epidural abscess and a left interhemispheric subdural empyema. The patient underwent emergency endoscopic sinus surgery (ESS) and neurosurgical drainage, followed by six weeks of antibiotic treatment, which resulted in full recovery with no neurological sequelae. One of the roles of ESS is to identify causative organisms, which can help guide antibiotic therapy and neurosurgical drainage. This case highlights the importance of early recognition and prompt surgical intervention in patients with invasive sinusitis complicated by intracranial complications.

## Introduction

Although pediatric rhinosinusitis typically resolves with conservative treatment owing to advances in antimicrobial therapy, it can occasionally result in serious complications such as intracranial and orbital complications [1]. Orbital abscesses may cause symptoms such as diplopia and visual impairment, which can persist even after treatment. Therefore, a prompt and appropriate diagnosis and intervention are essential. Pediatric intracranial complications are associated with high morbidity and mortality rates, making a timely diagnosis and management crucial [2]. However, the guidelines for extra-nasal complications following pediatric acute rhinosinusitis have not yet been established. In uncomplicated acute rhinosinusitis, *Streptococcus pneumoniae* and *Streptococcus pyogenes* are common bacterial pathogens. In contrast, *Streptococcus intermedius*, a member of the *Streptococcus anginosus* group (SAG), is a commensal organism of the oral cavity and upper respiratory tract, but is well known for its strong tendency to form abscesses [3]. Notably, *Streptococcus *species are the most common causative bacteria in pediatric intracranial complications of rhinosinusitis, and patients from whom this organism has been isolated tend to require more than one surgical procedure, such as craniotomy and endoscopic sinus surgery (ESS) [4,5]. Although such intracranial complications are uncommon, they are clinically significant and can spread through direct sinus wall extension, retrograde thrombophlebitis, or hematogenous routes [6]. We encountered a patient with both a frontal epidural abscess and an interhemispheric subdural empyema following acute rhinosinusitis caused by *Streptococcus intermedius*. Detailed clinical descriptions of such cases remain limited, making this report clinically informative. This case highlights the importance of early recognition and management of *S. intermedius*-associated complications in pediatric patients and contributes to the growing body of literature on intracranial complications following acute sinusitis in children.

## Case presentation

**Figure 1:**
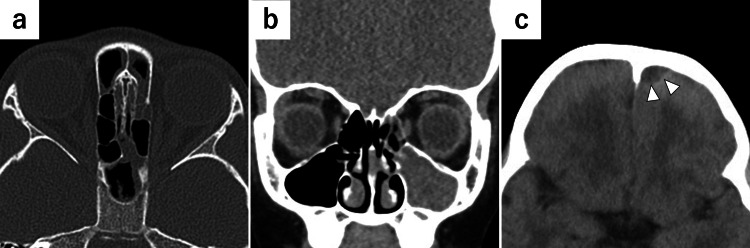
Non-contrast CT images Non-contrast CT images showing (a: axial, b: coronal) soft tissue opacification of the left paranasal sinuses and (c: axial) a low-density area in the left frontal region. No bone defects were observed. CT: computed tomography

A seven-year-old girl presented to a nearby clinic with fever and headache that lasted 10 days following nasal discharge. The patient had no specific medical history (including ENT or dental history). Laboratory investigations indicated leukocytosis (20,360/µL; normal: 4,000-8,000/µL), and the C-reactive protein (CRP) level was 8.9 mg/dL (normal: <0.5 mg/dL). These findings suggested the need for further evaluation and administration of intravenous antibiotic therapy; consequently, the patient was referred to a general hospital and diagnosed with bacterial pharyngitis. Ampicillin (166 mg/kg/day) was administered intravenously for three days, followed by cefotaxime (166 mg/kg/day) for four days. Although blood tests revealed a decrease in the CRP level from 8.9 to 3.2 mg/dL, the fever and headache persisted. Blood cultures revealed the presence of *Streptococcus intermedius* infection. Detailed documentation of the initial ENT and neurological examinations at the referral hospital was limited. Because the fever and headache persisted, a non-contrast computed tomography (CT) scan was performed on day 7 of hospitalization, which revealed a low-density area in the left frontal region and opacification of the ipsilateral paranasal sinus, with no bone defects in the left frontal sinus (Figure 1).

**Figure 2:**
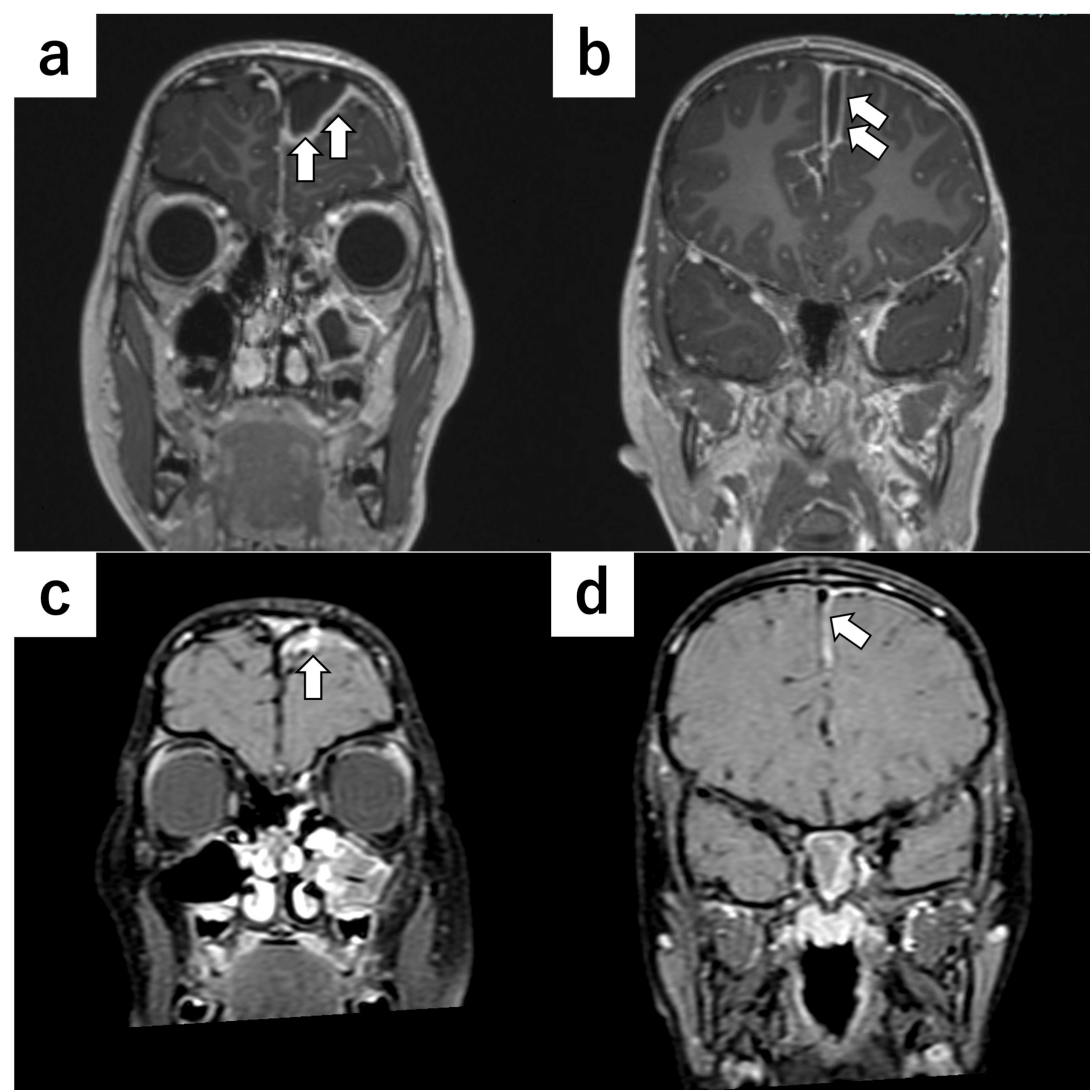
Coronal MRI of the patient with parasagittal frontal epidural abscess and interhemispheric subdural empyema Preoperative gadolinium-enhanced T1-weighted images (a: epidural abscess, b: subdural empyema) show rim-enhancing low-signal-intensity lesions, while postoperative fat-suppressed gadolinium-enhanced T1-weighted images obtained 19 days after surgery (c: epidural abscess, d: subdural empyema) demonstrate a marked reduction of both lesions. MRI: magnetic resonance imaging

On the same night, the patient experienced convulsions and was transferred to our hospital the following day. Contrast-enhanced magnetic resonance imaging (MRI) revealed a left parasagittal frontal epidural abscess and a left interhemispheric subdural empyema (Figure 2a, 2b). The presence of intracranial complications and neurological deterioration indicated the need for urgent surgical intervention. On the day of admission, the patient underwent a combined neurosurgical and endoscopic procedure, including drainage of the left parasagittal frontal epidural abscess and the left interhemispheric subdural empyema. A mini-craniotomy was performed using an endoscope to evacuate the subdural empyema. Pus was observed in both the maxillary and frontal sinuses, and mucosal edema was observed throughout the paranasal sinuses. Postoperatively, ceftriaxone (110 mg/kg/day) and metronidazole (30 mg/kg/day) were administered intravenously to enhance the central nervous system penetration and coverage for potential anaerobic co-infections commonly associated with *S. intermedius*. Cultures obtained from the pus during surgery revealed the presence of *Streptococcus intermedius*, and antibiotic susceptibility testing demonstrated sensitivity to ampicillin, cefotaxime, and ceftriaxone (Table 1). Based on these results, therapy was narrowed to intravenous ampicillin (400 mg/kg/day). Although adjustment of the antibiotics was necessary due to the emergence of a drug rash, the patient's headache and fever improved, and an MRI confirmed a reduction in the size of the epidural abscess and subdural empyema (Figure 2c, 2d). The patient was discharged after four weeks of intravenous antibiotic therapy, after which she completed an additional two weeks of oral antibiotics and has remained in remission since treatment.

**Table 1:** Antibiotic susceptibility profile of Streptococcus intermedius isolated from the abscess

Antibiotic	Result
Ampicillin	Sensitive
Cefotaxime	Sensitive
Ceftriaxone	Sensitive
Meropenem	Sensitive
Clindamycin	Sensitive
Metronidazole	Not applicable

**Figure 3:**
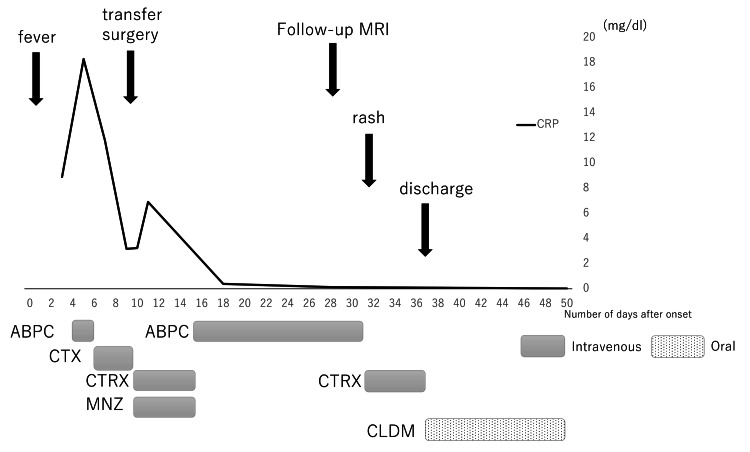
Case timeline Clinical course of the patient from symptom onset CRP (mg/dL) is plotted on the y-axis, and days from symptom onset on the x-axis. Colored bars below the x-axis indicate periods of antibiotic therapy (ABPC, CTX, CTRX, MNZ, and CLDM). Arrows indicate clinical events: surgery, follow-up MRI, appearance of rash, and discharge. CRP: C-reactive protein, ABPC: ampicillin, CTX: cefotaxime, CTRX: ceftriaxone, MNZ: metronidazole, CLDM: clindamycin, MRI: magnetic resonance imaging

The timeline of the case is presented in Figure 3. 

## Discussion

Pediatric acute rhinosinusitis rarely leads to intracranial complications; however, even with multidisciplinary treatment, mortality rates range from 3.3% to 9.8% [2,7]. Therefore, appropriate and timely treatment is crucial. Intracranial complications typically originate from frontal sinus infections, which spread through structural defects or the valveless diploic vein system via the bloodstream [6]. Patients most often present with nonspecific symptoms, such as headache, fever, nausea, and vomiting, and may not exhibit any neurological manifestations. Consequently, the diagnosis can be challenging and requires consideration of this rare condition [7]. Sekiyana et al. proposed the following seven criteria for suspected nasal intracranial complications: persistent fever; severe headache; nausea and vomiting; complications of orbital cellulitis; presence of Pott's puffy tumor; lesions in the frontal, ethmoid, or maxillary sinuses with rapidly worsening symptoms; and male sex during adolescence [8]. If at least one of the criteria is present, further examinations should be performed. In this patient's case, it took two weeks from the onset of headache to establish the diagnosis. Persistent fever and severe headache were observed during the initial visit. Therefore, imaging studies should have been performed early to evaluate possible intracranial complications.

Although the most common bacterial pathogens implicated in acute rhinosinusitis are *Streptococcus pneumoniae*, *Haemophilus influenzae*, and *Moraxella catarrhalis*, the predominant causative pathogen of intracranial abscesses following acute rhinosinusitis is *S. intermedius* [3,9]. This bacterium is a member of the *Streptococcus milleri* group and is typically a commensal microorganism found in the normal flora of the oral cavity and upper respiratory tract. *Streptococcus intermedius* is considered an important pathogen in cases of acute rhinosinusitis with intracranial complications, as affected patients often require surgical intervention despite the organism's relatively good in vitro susceptibility to antibiotics [3,4].

Several case reports and series have described pediatric patients with intracranial complications caused by *S. intermedius* or the *Streptococcus anginosus *group (SAG). In a recent pediatric cohort [5], SAG-related intracranial infections were more likely to require surgical intervention, particularly neurosurgical procedures, than infections caused by other pathogens. These reports, along with this case, highlight the aggressive nature of *S. intermedius* infections in children and the importance of early recognition and intervention.

The patient was administered intravenous ampicillin and cefotaxime, and the cultures showed good sensitivity to these antibiotics. Nevertheless, surgical drainage was necessary, which may be attributed to the organism's ability to form biofilms and its tendency to proliferate synergistically with anaerobic bacteria [10]. As *S. intermedius* has a well-recognized propensity to form rapidly progressive abscesses, its identification further strengthens the indication for early surgical drainage, in accordance with the general principle that most abscesses respond poorly to antibiotics alone.

There are no guidelines for the treatment of intracranial complications secondary to sinusitis. ESS is a relatively safe and minimally invasive procedure, and Kou et al. suggested that early ESS may reduce the need for subsequent neurosurgical intervention [11]. Bandino et al. have reported that ESS plays a key role in obtaining microbiological samples to guide targeted antimicrobial therapies [12]. However, concerns have been raised regarding the potential impact of ESS on facial growth in children, mainly originating from previous animal studies [13]. However, recent systematic reviews have concluded that most post-ESS evidence shows no significant differences in objective anthropometric measurements or subjective facial symmetry [14]. Based on our experience with this case, we believe that endoscopic sinus surgery (ESS) may be considered in selected patients with intracranial complications secondary to acute rhinosinusitis. The need for neurosurgical drainage is generally determined by the size of the abscess and the presence or absence of neurological symptoms [12].

Various empirical intravenous antibiotic regimens have been developed. The most frequently prescribed drugs were ceftriaxone/cefotaxime and metronidazole (63.4%) [15]. Otto et al. recommended the use of vancomycin, ceftriaxone, and metronidazole as empirical antibiotic regimens, with the discontinuation of vancomycin once the microbiological data showed no evidence of resistance to third-generation cephalosporins [16]. Although the duration of antibiotic treatment has not been determined, four to six weeks of antibiotic administration have resulted in good outcomes [15,16]. In this case, we administered ceftriaxone and metronidazole intravenously; vancomycin was not administered because *Streptococcus intermedius* was detected in the blood cultures. The total duration of antibiotic treatment was six weeks post-surgery, and the patient exhibited full recovery without any sequelae.

**Figure 4:**
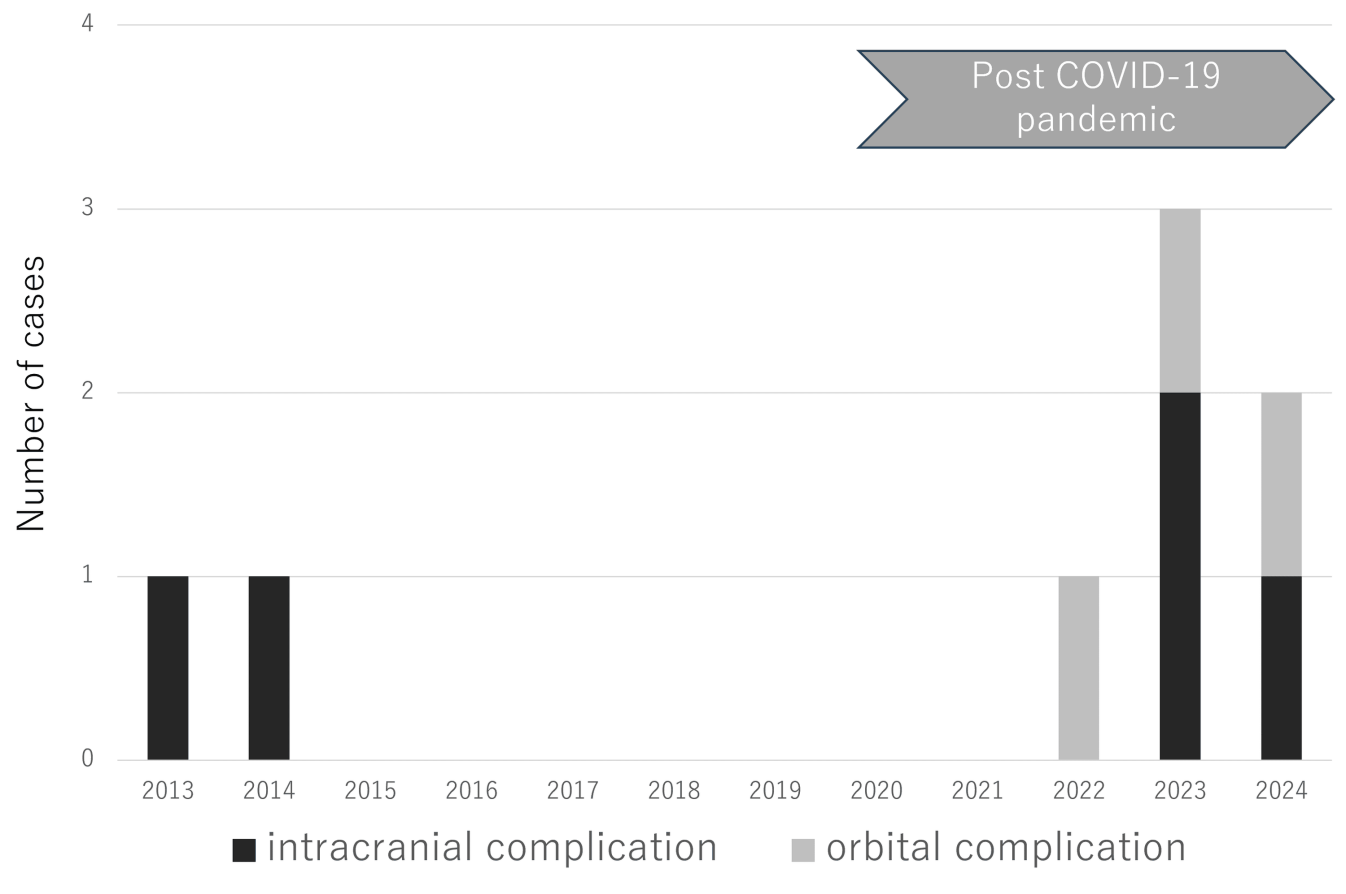
Trend in the number of pediatric cases of acute sinusitis with complications over the past three years COVID-19: coronavirus disease 2019 Image credit: Takato Sudo

Since the onset of the coronavirus disease 2019 (COVID-19) pandemic, several reports have noted an increase in pediatric cases of acute sinusitis with complications [17-19]. In our department, although the number of cases was limited, we observed an increase in the number of pediatric patients presenting with acute rhinosinusitis and its associated complications over the past three years (Figure 4). Although further studies are needed, one possible explanation is that reduced interpersonal contact during the pandemic may have altered children's immune responses. Because this trend may persist, the development of treatment guidelines is warranted.

## Conclusions

Here, we report the case of a pediatric patient with intracranial complications following acute rhinosinusitis. Prompt imaging is recommended when intracranial involvement is suspected based on symptoms and the clinical course. In such cases, ESS may facilitate the timely identification of the causative pathogen, enabling more effective treatment and quicker recovery. This case highlights the importance of early ESS and the clinical features of an *S. intermedius* infection in intracranial abscesses. However, as this report describes only a single case, caution is warranted when extrapolating these findings to other patients.
